# Letter to the Editor: Relationship of Choroidal Vasculature and Choriocapillaris Flow With Alterations of Salivary α-Amylase Patterns in Central Serous Chorioretinopathy

**DOI:** 10.1167/iovs.63.6.11

**Published:** 2022-06-09

**Authors:** Matteo Menean, Riccardo Sacconi, Giuseppe Querques

**Affiliations:** 1 Department of Ophthalmology, University Vita-Salute, IRCCS Ospedale San Raffaele, Milan, Italy. E-mail: giuseppe.querques@hotmail.it, querques.giuseppe@hsr.it.

We read with great interest the observational study by Scarinci et al. on the relationship between choroidal flow and salivary α-amylase (α-AMY) secretive patterns in central serous chorioretinopathy (CSC).^1^ This study provides evidence for a putative role of autonomous system dysfunction as a cause of choroidal flow in patients with CSC, namely a flattened diurnal percentage variation of salivary α-AMY correlates with increased choroidal vascularity index, subfoveal choroidal thickness, and flow signal void area.

The authors used reliable and standardized methods and timing for salivary α-AMY collection to reduce the considerable heterogeneity of α-AMY levels.^2^ Although they used this robust methodological approach, many determinants cannot be controlled, first, the parasympathetic drive in salivary flow regulation.^3^^,^^4^ Furthermore, the physiological pattern of salivary α-AMY levels has a sudden decrease upon awakening, with a minimum peak approximately 30 minutes after waking up and a progressive increase during the day.^4^ That being so, an additional sampling immediately after waking up and 30 minutes later could provide more precise quantification of the area under the curve of salivary α-AMY diurnal production. Most importantly, the study sets the α-AMY levels sampled 60 minutes after awaking as baseline for the diurnal percentage variation between morning and evening: a sampling at 0, 30, and 60 minutes after awaking better identifies the lowest value of α-AMY levels and allows a more accurate quantification of the enzyme secretive pattern and percentage variation.

Moreover, according to the idea of an autonomic dysfunction, the authors addict an increased sympathetic drive as a cause of the abnormalities of α-AMY levels pattern presented in the study. However, the relationship between the levels of salivary α-AMY and sympathetic activation is still ambiguous.^2^^,^^3^ Although an increase in α-AMY levels has been reported after acute stress,^5^ chronic stressful stimuli do not barely cause an increase of sympathetic activity, but a set of hormonal imbalances. In addition, contrasting data suggest a reduced α-AMY levels or preserved diurnal fluctuation after chronic stress.^6^^–^^8^ We should not therefore consider α-AMY levels alteration in patients with CSC as a manifestation of an increased sympathetic drive, but rather as evidence of a hormonal and autonomous imbalance, which is still only partially understood.

Referring to a previous work of the same group,^9^ patients with CSC presented a significant alteration of α-AMY diurnal fluctuation, with no different levels in the morning respect to the control group, but higher levels in the evening. These results seem to contrast with the lower diurnal percentage variation of salivary α-AMY levels. This consideration highlights the complexity of hormonal changes in patients with CSC and the limits of salivary α-AMY sampling, as well as the challenging interpretation of salivary α-AMY levels’ alteration. Increasing evidence supports the imbalance between the mineralocorticoid and glucocorticoid patterns as a pathogenetic mechanism for CSC.^10^ This complex framework involves salivary α-AMY, but the mechanisms are still partially unexplained.

According to what has been said, we were wondering if the authors may provide an interpretation of their data at the light of the aforementioned comments, with particular reference to the results of their previous study.^9^
